# Restoration of foveal photoreceptors after intravitreal ranibizumab injections for diabetic macular edema

**DOI:** 10.1038/srep39161

**Published:** 2016-12-14

**Authors:** Yuki Mori, Kiyoshi Suzuma, Akihito Uji, Kenji Ishihara, Shin Yoshitake, Masahiro Fujimoto, Yoko Dodo, Tatsuya Yoshitake, Yuko Miwa, Tomoaki Murakami

**Affiliations:** 1 Department of Ophthalmology and Visual Sciences, Kyoto University Graduate School of Medicine, Kyoto, Japan

## Abstract

Anti-vascular endothelial growth factor drugs are the first-line treatment for diabetic macular edema (DME), although the mechanism of the visual acuity (VA) improvement remains largely unknown. The association between photoreceptor damage and visual impairment encouraged us to retrospectively investigate the changes in the foveal photoreceptors in the external limiting membrane (ELM) and ellipsoid zone (EZ) on spectral-domain optical coherence tomography (SD-OCT) images in 62 eyes with DME treated with intravitreal ranibizumab (IVR) injections. The transverse lengths of the disrupted EZ and ELM were shortened significantly (*P* < 0.001 and *P* = 0.044, respectively) at 12 months. The qualitative investigation also showed restoration of the EZ and ELM lines on SD-OCT images. The EZ at 12 months lengthened in 34 of 38 eyes with discontinuous EZ and was preserved in 16 of 21 eyes with complete EZ at baseline. VA improvement was positively correlated with shortening of the disrupted EZ at 12 months (*ρ* = 0.463, *P* <* *0.001), whereas the decrease in central subfield thickness was associated with neither VA improvement nor changes in EZ status (*ρ* = 0.215, *P* = 0.093 and (*ρ* = 0.209, *P* = 0.103, respectively). These data suggested that photoreceptor restoration contributes to VA improvement after pro re nata treatment with IVR injections for DME independent of resolved retinal thickening.

Diabetic retinopathy (DR) is a leading cause of visual loss in patients of working age, and proliferative diabetic retinopathy (PDR) and diabetic macular edema (DME) especially often threaten vision in patients with diabetes^1^,^2^. Diabetes promotes disruption of the blood-retinal barrier and deteriorates neuroglial function, although it remains to be elucidated how extravasated blood constituents exacerbate dysfunction of the individual neuroglial components in DME^3^,^4^,^5^.

Vascular endothelial growth factor (VEGF) has multiple pathological and physiological effects, i.e., angiogenesis, vascular hyperpermeability, induction of DR-like vascular changes, prothrombotic or antithrombotic responses, and neuroprotection^6^,^7^,^8^,^9^,^10^,^11^,^12^. Both molecular and clinical investigations have allowed clinicians to administer anti-VEGF therapy to treat DME and improve visual prognoses. This first-line treatment reduces retinal vascular permeability and retinal thickness and has beneficial effects on DR progression, deposition of hard exudates, and the nonperfused areas in the macula^13^,^14^,^15^,^16^,^17^,^18^. Despite accumulating evidence regarding the efficacy on the retinal vasculature, it remains ill-defined how anti-VEGF drugs restore the neuronal components.

A recent study reported extensive production of reactive oxygen species in the photoreceptors in diabetic retinas^19^. Advances in spectral-domain optical coherence tomography (SD-OCT) technology have increased the understanding of morphologic changes in individual retinal layers and their association with molecular mechanisms in chorioretinal diseases^20^,^21^. The photoreceptor status especially is represented by the ellipsoid zone (EZ) of the photoreceptors and the external limiting membrane (ELM), and disruption of these lines is related to visual impairment in macular edema in retinal vascular diseases including DME and retinal degenerative diseases^22^,^23^,^24^,^25^,^26^,^27^,^28^,^29^,^30^. A few studies have shown these biomarkers of the photoreceptor status as predictors of visual outcomes after anti-VEGF therapy^31^,^32^,^33^. However, the effects of anti-VEGF therapy on photoreceptor damage are controversial^34^,^35^,^36^.

In the current study, we qualitatively and quantitatively investigated the morphologic changes in the foveal photoreceptor markers, i.e., the EZ and ELM, after pro re nata (PRN) treatment with intravitreal ranibizumab (IVR) injections ((Lucentis^®^; Novartis Pharma AG, Basel, Switzerland; Genentech Inc., South San Francisco, CA, USA) for DME and the association with visual acuity (VA) improvements.

## Results

### Photoreceptor restoration after IVR injections

We reviewed 62 eyes of 58 patients with center-involved DME treated with IVR injections. Among 118 eyes that met the eligible criteria, nine eyes were excluded at baseline. Forty-seven of the 109 included eyes were lost to follow-up before the 12-month examination. According to 3+ PRN regimen, 62 eyes received six (interquartile range [IQR], 4–9) injections for 12 months, and the logarithm of the minimum angle of resolution (logMAR) VA and central subfield (CSF) thickness improved from 0.260 (0.155–0.506) and 441 μm (397–524) at baseline to 0.155 (0.046–0.281) and 300 μm (268–379) at 12 months. The baseline systemic and ocular characteristics are shown in Table 1.

**Figure 1:**
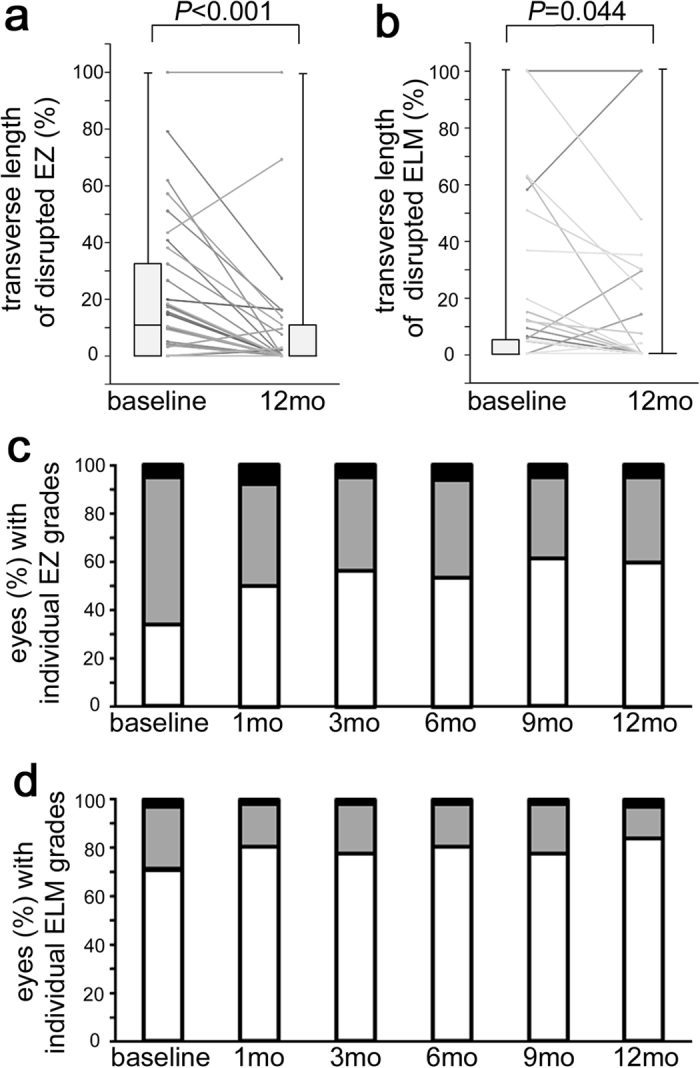
Morphologic restoration of foveal photoreceptors after IVR for DME. The shortening in the transverse length of the disrupted EZ (**a**) and disrupted ELM (**b**) on SD-OCT images at 12 months. (**c,d**) The qualitative changes in the EZ and ELM after IVR for DME. White bar = complete; gray bar = discontinuous; black bar = absent.

We evaluated the qualitative and quantitative changes in the photoreceptor status in 62 eyes with center-involved DME treated with IVR injections. The transverse length of the disrupted EZ at the fovea shortened significantly at 12 months (10.4% [0–31.6] vs. 0% [0–10.5], *P* < 0.001) (Fig. 1a), and the transverse length of the disrupted ELM also decreased significantly (*P* = 0.044) (Fig. 1b). Qualitative investigation showed that the number of eyes with the complete EZ gradually increased from 21 (33.9%) eyes at baseline to 37 (59.7%) eyes at 12 months compared to the decreased number of eyes with the discontinuous EZ, i.e., 38 (61.3%) eyes at baseline to 22 (35.5%) eyes at 12 months (Fig. 1c, Table 2). SD-OCT showed the complete ELM at the fovea in 44 and 52 eyes at baseline and 12 months, respectively (Fig. 1d, Table 2).

**Figure 2:**
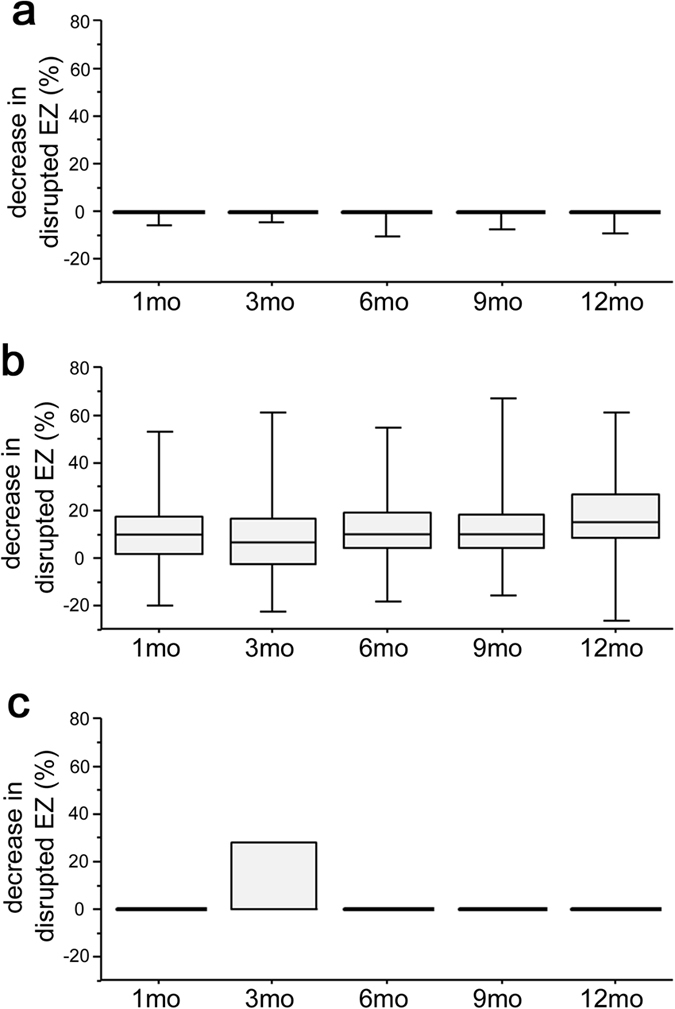
Individual changes in the foveal EZ after IVR in eyes with individual EZ status at baseline. Decrease in the transverse length of disrupted EZ at 12 months in 21 eyes of the complete EZ group (**a**), 38 eyes of the discontinuous EZ group (**b**), and three eyes of the absent EZ group (**c**) at baseline.

**Figure 3:**
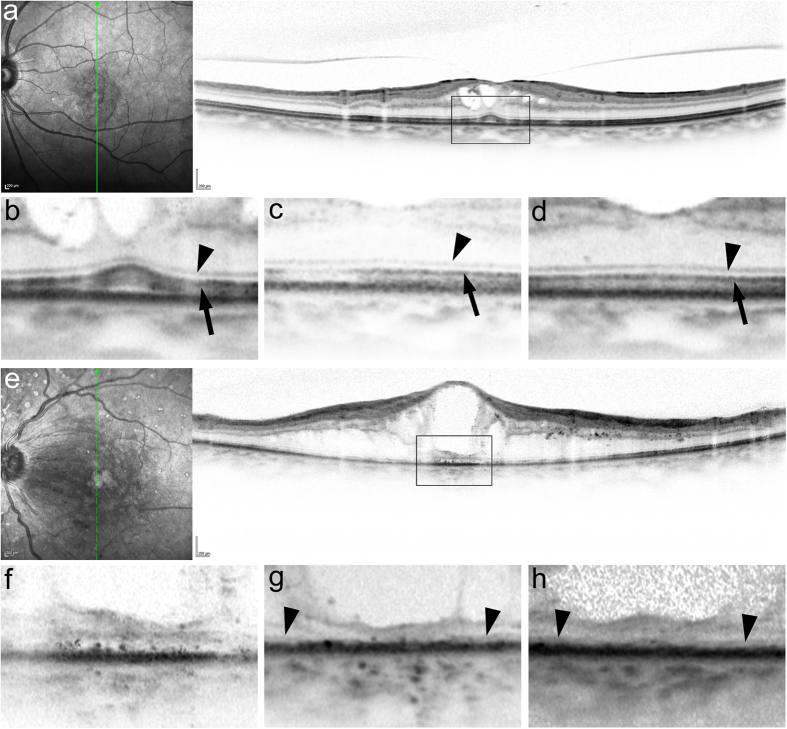
Absence of marked changes in the foveal photoreceptor integrity after IVR in representative cases with the complete or absent EZ at baseline. (**a,b**) SD-OCT images at baseline show the complete EZ and ELM at the fovea in a 74-year-old patient with DME treated with six IVR injections. Baseline logMAR VA = 0.222. (**c,d**) These lines are preserved throughout 12 months. LogMAR VA at 12 months = 0. (**e,f**) Preoperative SD-OCT images show an absent EZ and ELM at the fovea in a 57-year-old patient with DME. Baseline logMAR VA = 1. (**g,h**) The foveal EZ is not seen during the 12-month follow-up during which the patient received 10 injections. LogMAR VA at 12 months = 0.824. (**b,f**) The magnified images in the black rectangle in a and e. The EZ and ELM at 3 (**c,g**) and 12 months (**d,h**). Arrowheads = ELM. Arrows = EZ.

**Figure 4:**
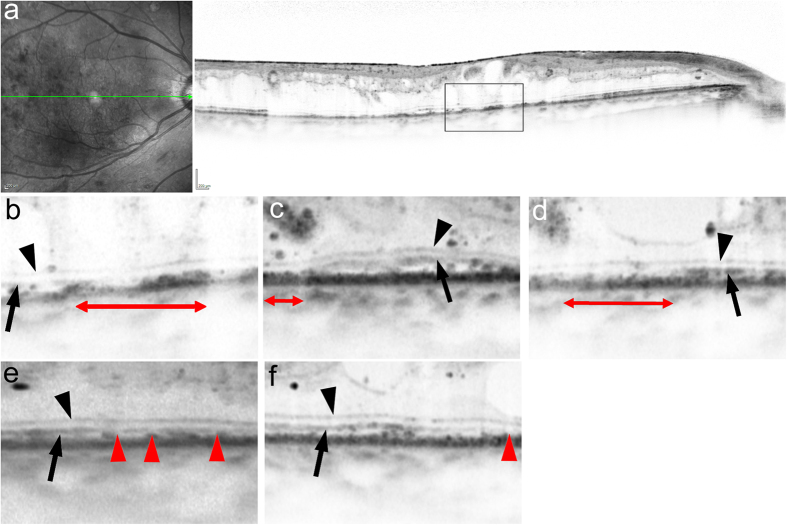
Morphologic restoration of foveal photoreceptors after IVR in a representative case with the discontinuous EZ at baseline. (**a,b**) Horizontal retinal sections at baseline show a discontinuous EZ in a 60-year-old patient with DME of the cystoid macular edema type who was treated with 10 IVR injections. Baseline logMAR VA = 0.398. The foveal EZ is restored partially at 1, 3, and 6 months (**c,d,e**) and almost completely at 12 months (**f**). LogMAR VA at 12 months = 0.046. Black arrowheads = ELM. Black arrow = EZ. Red arrowheads or double-headed arrows = areas without an EZ.

Of 21 eyes of the complete EZ group at baseline, 16 (76.2%) eyes had preserved intact EZ throughout the 12 months of follow-up, and only three (14.3%) eyes had the discontinuous EZ at 12 months (Figs 2a and 3a–d). The disappearance of the EZ line persisted in all three eyes with the absent EZ at baseline (Figs 2c and 3e–h). In contrast, the transverse length of the disrupted EZ decreased in most eyes (89.5%) with the discontinuous EZ (19.4% [10.4–42.8] and 1.3% [0.0–14.4] at baseline and 12 months, respectively) (Figs 2b and 4). Further, the decrease in the transverse length of the disrupted EZ at 12 months was correlated with the logMAR VA and the transverse length of the disrupted EZ and ELM at baseline, whereas there was no association with preoperative systemic parameters (Table 3). The shortening of the disrupted ELM at 12 months was associated with the baseline logMAR VA, CSF thickness, and the transverse length of the disrupted EZ and ELM (Table 4).

### Association between VA improvement and photoreceptor restoration

**Figure 5:**
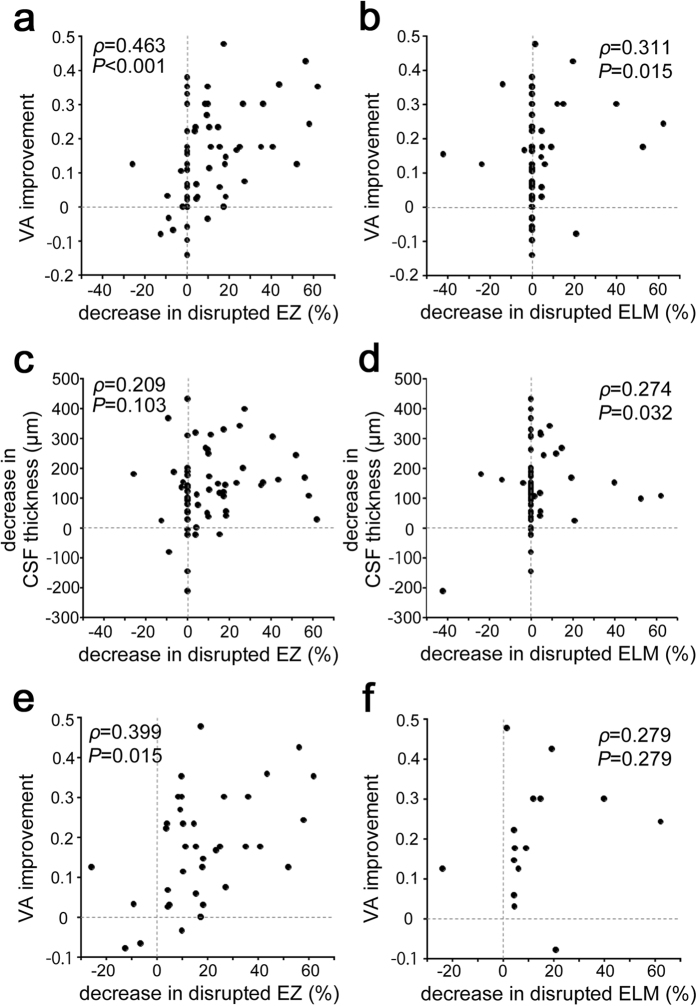
Relationship between shortening of the disrupted EZ and VA improvement after IVR for DME. VA improvement is associated with a decrease in the transverse length of the disrupted EZ (**a**) or disrupted ELM (**b**) at 12 months in all 62 eyes. The relationship between the decreases in CSF thickness and the transverse length of the disrupted EZ (**c**) or disrupted ELM (**d**) at 12 months in all 62 eyes. (**e**) In 38 eyes with the discontinuous EZ at baseline, the decrease in the disrupted EZ is related to VA improvement at 12months. (**f**) There is no correlation between the VA improvement and the decrease in the disrupted ELM in 16 eyes with the discontinuous ELM at baseline.

A positive association was seen in all 62 eyes between the VA improvements and the decrease in the transverse length of the disrupted EZ or ELM at 12 months (*ρ* = 0.463, *P* < 0.001 or *ρ* = 0.311, *P* = 0.015, respectively; Fig. 5a,b), whereas there was no correlation between the changes in the logMAR VA and CSF thickness (*ρ* = 0.215, *P* = 0.093). Further multiple regression analysis with a forward stepwise approach also confirmed the association between VA improvement and the decrease in transverse length of the disrupted EZ (*β* = 0.438, *P* < 0.001). The VA improvement was related to the decreased disruption of the EZ in 38 eyes with the discontinuous EZ at baseline (*ρ* = 0.399, *P* = 0.015) (Fig. 5e). These data might be consistent with the association between the disrupted EZ or ELM and the logMAR VA at 12 months (Table 5). Further statistical analyses showed that the transverse length of the disrupted EZ or ELM at baseline was related to poorer visual outcomes and greater efficacy after IVR injections (Tables 6 and 7).

## Discussion

Despite the definite efficacy of anti-VEGF drugs on VA, it remains largely unknown how anti-VEGF therapy ameliorates the neuroglial dysfunction in DME. In the current study, the quantitative investigation showed restoration of the foveal photoreceptors and the association with improved VA after administration of IVR injections to treat DME. The EZ status especially improved in most eyes with the discontinuous EZ at baseline and was preserved in most eyes with the complete EZ at baseline for the 1-year follow-up. These data suggested a novel therapeutic target for anti-VEGF therapies in the neuronal components and well-known targets in the retinal vascular lesions^16^,^17^,^18^. In addition, clinicians often see better VA when the macular edema recurs during PRN treatments, which might be explained partly by photoreceptor restoration after IVR injections. It suggested that the photoreceptor integrity could be a novel surrogate marker to evaluate the efficacy of IVR treatment with as-needed regimen.

The EZ, previously known as the third high-reflectance band or the junction between the inner and outer segments (IS/OS), clinically represents the photoreceptor integrity in several diseases^22^,^24^,^25^,^26^. The biological EZ is comprised mainly of mitochondria and enables higher levels of energy consumption in the photoreceptors. The absence of the EZ on SD-OCT images might correspond to the anatomic absence or the reduced OCT reflectivity of the EZ. This led us to hypothesize that the mitochondrial dysfunction in the foveal photoreceptors leads to reduced VA in DME. Although it remains ill-defined what blood constituents exacerbate photoreceptor damage, anti-VEGF treatment might block their extravasation with concomitant restoration of the EZ and outer segments on SD-OCT images^34^,^37^,^38^. In addition, anti-VEGF therapy might resolve foveal cystoid spaces, which seem to compress and deform the photoreceptor layers^25^.

The ELM is the junctional complex between the Müller and photoreceptor cells and has barrier properties against macromolecules^39^,^40^. The disrupted ELM might allow blood constituents to pour into the subretinal spaces and damage the photoreceptors, or vice versa, photoreceptor damage might lead to disruption of the ELM in DME^37^. The disrupted ELM was shortened modestly after anti-VEGF therapy, which caused speculation that blocking the neurotoxic blood constituents promotes restoration of the photoreceptors and shortening of the disrupted ELM reciprocally.

The statistical analyses suggested that the decrease in the disrupted EZ contributed to VA improvement independent of the reduced retinal thickness after IVR injections for DME (Fig. 5a,c). In contrast, modest associations were seen between the disrupted ELM and CSF thickness at baseline and 12 months and between decreases in the disrupted ELM and CSF thickness. Two possible explanations should be considered, i.e., damage in the Müller cells might contribute to both ELM disruption and inner retinal thickening^39^,^41^, and the disrupted barrier properties in the ELM might dysregulate the fluid dynamics and concomitantly allow accumulation of intraretinal or subretinal fluids^42^.

Generally, when neurons degenerate, they do not regenerate, as Ramon y Cajal proposed^43^. In contrast, many publications have reported neuronal regeneration in several situations^44^. We could not determine whether the morphologic restoration of the ELM and EZ on the SD-OCT images corresponded to regeneration of the photoreceptor cells. Most eyes with DME in which the ELM or EZ were disrupted have an outer nuclear layer (ONL) just above these lesions, although the ONL is comprised of the cellular bodies of the photoreceptor cells connected to the inner and outer segments^26^. This inconsistency suggests subcellular damage rather than complete cellular death as in synaptic pruning in neurons^45^. Thus, we might speculate that anti-VEGF treatment blocks neurotoxic blood constituents and allows restoration of the inner and outer segments from the surviving cell bodies of the photoreceptor cells in the ONL. It might be analogous to the disappearance and restoration of the EZ rather than the ONL in the acute zonal occult outer retinopathy complex compared to the loss of any photoreceptor layers in retinal degenerative diseases^22^,^46^,^47^. In contrast, three eyes with the absent EZ at baseline did not have improvements in the EZ status at 12 months and might share the common mechanisms with retinal degenerative diseases. However, another clinicopathological study using SD-OCT images should reveal the histologic correspondence in different diseases.

Among several therapeutic strategies, vitrectomy might be efficacious in eyes with DME and vitreomacular traction^48^. In addition, vitreoretinal abnormalities on OCT images and surface wrinkling retinopathy on color fundus photograph are factors predictive of poorer visual outcomes and efficacy after administration of IVR injections to treat DME^49^. A recent study showed that the number of eyes with the disrupted ELM increased from 37.5% at baseline to 56.3% at the final visit after vitrectomy, whereas the surgery allows clinicians to remove vitreomacular traction^50^. In this study, IVR decreased eyes with the disrupted ELM (from 29.0% at baseline to 16.1% at 12 months), but cannot treat the lesions at the vitreoretinal interface. These data might allow consideration of customized medicine to treat DME.

This retrospective study had limitations, with the first being the small number of cases. We applied the subjective quantification of photoreceptor damage, although the automatic measurements using image processing procedures generally improve the reproducibility and objectivity^51^,^52^,^53^. Compared to the degenerative processes of photoreceptors in geographic atrophy or retinitis pigmentosa, photoreceptor damage in DME is often accompanied with hyperreflective foci^38^. The automatic methods cannot completely discriminate such lesions from the fragmented EZ lines and therefore did not measure the EZ lines precisely. The images of the outer retinal layers often were affected by the hyperreflective lesions in the inner retinal layers, which suggested that the changes in the ELM and EZ might sometimes be artifacts. The modest correlation between the disrupted ELM and CSF thickness suggested that the inner retinal edema might block the faint reflective signals of the ELM (Table 7). Another study should determine if these results are generalizable to other populations, injection regimens, and imaging instruments.

In conclusion, we showed the beneficial effects of IVR treatment on foveal photoreceptor damage in DME, which suggested one of multiple mechanisms in VA improvement associated with this treatment and shed light on the path to customized medicine for patients with DME.

## Methods

### Participants

We retrospectively investigated the changes in foveal photoreceptor status at 12 months in 62 consecutive eyes of 58 patients with center-involved DME treated with IVR injections for 12 months or longer^54^. Patients with center-involved DME who visited Kyoto University Hospital from March 2014 to April 2015 as the baseline visit received IVR injections according to the 3+ PRN regimen. The exclusion criteria at baseline were media opacities affecting the VA, other chorioretinal diseases, treatment for DME within the previous 6 months, previous intraocular surgery other than cataract extraction, and cataract surgery within the previous 3 months. Further, several patients dropped out during the 12-month follow-up, because of patient’s inconvenience, patient desire to terminate treatment or change to other therapeutic strategies, drug tachyphylaxis, or additional treatments, i.e., focal/grid photocoagulation, panretinal photocoagulation, vitrectomy (for vitreous hemorrhage), or cataract surgery. All research and measurements adhered to the tenets of the Declaration of Helsinki. The institutional review board and the ethics committee of Kyoto University Graduate School of Medicine approved the study protocol. All participants provided written informed consent before study enrollment.

### Intervention

Ranibizumab (0.5 mg) was injected intravitreally according to the 3+ PRN regimen described in the Ranibizumab Monotherapy or Combined with Laser versus Laser Monotherapy for Diabetic Macular Edema (RESTORE) study^14^. After disinfection, ranibizumab was injected 3.5 mm posterior to the limbus followed by instillation of antibiotics. Three monthly ranibizumab injections were followed by the PRN injections according to the retreatment criteria of the RESTORE study^14^.

### OCT

The best-corrected decimal VA was measured at all visits and converted to the logMAR VA for statistical analyses followed by comprehensive ophthalmic examinations. After calibration using the corneal curvature radii and refractive error, we obtained OCT images using SD-OCT (Spectralis OCT, Heidelberg Engineering, Heidelberg, Germany) at every monthly visit^55^. Vertical and horizontal sectional images dissecting the fovea were acquired using the cross-hair mode (30 degrees). This instrument has a high A-scan rate of 40,000 A-scans/second using a light source of approximately 870 nm, using the optical resolution of approximately 7 μm in depth (axial optical resolution) and 14 μm transversally (lateral optical resolution). Thirty-degree B-scan of the high resolution mode was composed of 1,536 A-scans with 3.5 μm digital axial resolution. Resultantly, the digital lateral resolution was 5.67 μm (5.52–5.81), which depended on the corneal curvature radii and refractive error in individual eyes. The number for image averaging was 20 to 100 to acquire better images. Three-dimensional images also were obtained using the raster scan mode, followed by the construction of two-dimensional maps and automatic quantification of the mean CSF thickness, as described previously^56^.

We qualitatively and quantitatively evaluated the foveal photoreceptor status using the two high-reflectance bands, EZ and ELM, on the vertical and horizontal sectional images at the monthly visits. We qualitatively categorized the status of these lines into complete, discontinuous, and absent as described previously^22^,^24^,^38^. Two retina specialists evaluated these markers of photoreceptor integrity. If they disagreed, a third specialist participated. We also quantified the transverse length of the disrupted EZ or ELM as previously reported^25^. Briefly, after the exclusion of the areas where reflectivity signals in the retinal pigment epithelium (RPE) were attenuated by media opacity or hyperreflective lesions in inner retinal layers, the areas with damaged photoreceptors were quantified. The EZ lines with various OCT reflectivity levels were divided into three categories; i.e., intact, faint, and disrupted. The ELM status was defined as intact or disrupted. We thus measured the transverse length of the areas where the EZ or ELM line was disrupted within the central 1 mm on the vertical and horizontal images using the caliper tool in the Heidelberg Eye Explorer software (Heidelberg Engineering). The average of the percentage was used in the following investigations.

### Statistical analysis

The results are expressed as the median (IQR). The data were analyzed using the Wilcoxon signed-rank test or the Mann-Whitney *U*-test to evaluate the differences between two groups. Spearman’s correlation coefficient was calculated to test the statistical correlation. We employed the multiple regression analysis with a stepwise forward approach (age, gender, the decrease in CSF thickness, and the decrease in the transverse length of the disrupted EZ as independent variables; VA improvement as a dependent variable). *P* < 0.05 was considered significant.

## Additional Information

**How to cite this article**: Mori, Y. *et al*. Restoration of foveal photoreceptors after intravitreal ranibizumab injections for diabetic macular edema. *Sci. Rep.* **6**, 39161; doi: 10.1038/srep39161 (2016).

**Publisher’s note:** Springer Nature remains neutral with regard to jurisdictional claims in published maps and institutional affiliations.

## Figures and Tables

**Table 1:** Baseline characteristics and their association with photoreceptor status.

Parameter		Association with the transverse length of disrupted EZ	Association with the transverse length of disrupted ELM
Eyes/patients	62/58	—	—
Age (years)	69 (59–74)	*ρ* = −0.048,*p* = 0.709	*ρ* = 0.142,*p* = 0.267
Gender		*p* = 0.616	*p* = 0.388
Men	32	14.6% (1.5–28.0)	0.0% (0.0–0.0)
Women	26	9.9% (0.0–40.7)	0.0% (0.0–5.4)
HbA1c	7.1% (6.7–7.6)	*ρ* = −0.197,*p* = 0.167	*ρ* = 0.029,*p* = 0.837
Systemic hypertension	21/37	*p* = 0.544	*p* = 0.419
Absent		5.0% (0.0–35.2)	0.0% (0.0–0.0)
Present		15.4% (0.0–26.4)	0.0% (0.0–4.6)
LogMAR VA	0.260 (0.155–0.506)	*ρ* = 0.513,*p* < 0.001	*ρ* = 0.591,*p* < 0.001
Lens status		*p* = 0.406	*p* = 0.233
Phakia	40 eyes	14.5% (0.0–37.2)	0.0% (0.0–10.2)
Pseudophakia	22 eyes	6.9% (0.0–30.1)	0.0% (0.0–0.0)
DR severity			
Mild NPDR	1 eye	0.0%	0.0%
Moderate NPDR	33 eyes	10.4% (0.0–19.9)	0.0% (0.0–0.0)
Severe NPDR	12 eyes	20.9% (3.8–56.3)	0.0% (0.0–13.5)
PDR	16 eyes	10.0% (3.2–58.6)	0.0% (0.0–21.7)
Panretinal photocoagulation		*p* = 0.012	*p* = 0.007
Absent	22 eyes	4.7% (0.0–19.9)	0.0% (0.0–0.0)
Present	40 eyes	16.3% (0.0–53.8)	0.0% (0.0–9.7)
CSF thickness	441 μm (397–524)	*ρ* = 0.218,*p* = 0.089	*ρ* = 0.321,*p* = 0.012
Cystoid macular edema		*p* = 0.499	*p* = 0.238
Absent	13 eyes	5.0% (0.0–56.1)	0.0% (0.0–19.3)
Present	49 eyes	14.4% (0.0–26.5)	0.0% (0.0–4.5)
Serous retinal detachment		*p* = 0.679	*p* = 0.907
Absent	43 eyes	14.6% (0.0–30.9)	0.0% (0.0–4.5)
Present	19 eyes	7.7% (0.0–35.0)	0.0% (0.0–2.7)

HbA1c = hemoglobin A1c; NPDR = nonproliferative diabetic retinopathy.

**Table 2:** Qualitative changes in photoreceptor status after intravitreal ranibizumab for DME.

EZ status at baseline	EZ status at 12 months		
	Complete EZ	Discontinuous EZ	Absent EZ
Complete EZ	18	3	0
Discontinuous EZ	19	19	0
Absent EZ	0	0	3
**ELM status at baseline**	**EZ status at 12 months**		
	**Complete ELM**	**Discontinuous ELM**	**Absent ELM**
Complete ELM	42	2	0
Discontinuous ELM	10	5	1
Absent ELM	0	1	1

**Table 3:** Association between baseline characteristics and disrupted EZ 12 months after ranibizumab for DME.

Parameter at baseline	Disrupted EZ at 12months	EZ improvement at 12 months
LogMAR VA	*ρ* = 0.423, *p* < 0.001	*ρ* = 0.269, *p* = 0.036
Lens status	*p* = 0.716	*p* = 0.439
Phakia	0.0% (0.0–12.9)	9.1% (0.0–17.3)
Pseudophakia	0.0% (0.0–7.8)	3.8% (0.0–17.4)
International classification		
Mild NPDR	0.0%	0.0%
Moderate NPDR	0.0% (0.0–6.0)	3.7% (0.0–17.3)
Severe NPDR	5.5% (0.0–16.9)	15.4% (3.8–38.0)
PDR	0.0% (0.0–16.8)	2.2% (0.0–10.0)
Panretinal photocoagulation	*p* = 0.067	*p* = 0.230
Absent	0.0% (0.0–1.9)	2.2% (0.0–16.5)
Present	0.0% (0.0–16.0)	6.8% (0.0–17.5)
CSF thickness	*ρ* = 0.303, *p* = 0.018	*ρ* = 0.014, *p* = 0.915
Transverse length of disrupted EZ	*ρ* = 0.689, *p* < 0.001	*ρ* = 0.642, *p* < 0.001
Transverse length of disrupted ELM	*ρ* = 0.797, *p* < 0.001	*ρ* = 0.353, *p* = 0.006
Cystoid macular edema	*p* = 0.662	*p* = 0.616
Absent	0.0% (0.0–16.9)	0.0% (0.0–36.1)
Present	0.0% (0.0–7.7)	8.6% (0.0–17.3)
Serous retinal detachment	*p* = 0.381	*p* = 0.731
Absent	0.0% (0.0–10.2)	4.4% (0.0–17.3)
Present	0.0% (0.0–9.7)	4.3% (0.0–21.8)

**Table 4:** Relationship between baseline characteristics and improvement in foveal ELM 12 months after ranibizumab for DME.

Parameter at baseline	Disrupted ELM at 12months	ELM improvement at 12 months
LogMAR VA	*ρ* = 0.534, *p* < 0.001	*ρ* = 0.456, *p* < 0.001
Lens status	*p* = 0.820	*p* = 0.063
Phakia	0.0% (0.0–0.0)	0.0% (0.0–4.5)
Pseudophakia	0.0% (0.0–0.0)	0.0% (0.0–0.0)
International classification		
Mild NPDR	0.0%	0.0%
Moderate NPDR	0.0% (0.0–0.0)	0.0% (0.0–0.0)
Severe NPDR	0.0% (0.0–7.1)	0.0% (0.0–4.5)
PDR	0.0% (0.0–0.0)	0.0% (0.0–13.8)
Panretinal photocoagulation	*p* = 0.069	*p* = 0.090
Absent	0.0% (0.0–0.0)	0.0% (0.0–0.0)
Present	0.0% (0.0–0.0)	0.0% (0.0–4.5)
CSF thickness	*ρ* = 0.350, *p* = 0.006	*ρ* = 0.274, *p* = 0.032
Transverse length of disrupted EZ	*ρ* = 0.673, *p* < 0.001	*ρ* = 0.463, *p* < 0.001
Transverse length of disrupted ELM	*ρ* = 0.777, *p* < 0.001	*ρ* = 0.794, *p* < 0.001
Cystoid macular edema	*p* = 0.560	*p* = 0.251
Absent	0.0% (0.0–0.0)	0.0% (0.0–0.0)
Present	0.0% (0.0–0.0)	0.0% (0.0–0.0)
Serous retinal detachment	*p* = 0.320	*p* = 0.476
Absent	0.0% (0.0–0.0)	0.0% (0.0–0.8)
Present	0.0% (0.0–0.0)	0.0% (0.0–0.0)

**Table 5:** Association between photoreceptor status and logMAR VA at 12 months.

	logMAR VA	CSF thickness
Transverse length of disrupted EZ	*ρ* = 0.392,*p* = 0.002	*ρ* = 0.089,*p* = 0.487
Transverse length of disrupted ELM	*ρ* = 0.492,*p* < 0.001	*ρ* = 0.353,*p* = 0.006

**Table 6:** Relationship between baseline characteristics and VA improvement after ranibizumab for DME.

Parameter at baseline	logMAR VA at baseline	logMAR VA at 12 months	VA improvement at 12 months
Age (years)	*ρ* = −0.092, *p* = 0.474	*ρ* = 0.076, *p* = 0.555	*ρ* = −0.131, *p* = 0.307
Gender	*p* = 0.481	*p* = 0.964	*p* = 0.144
Men	0.301 (0.155–0.456)	0.125 (0.046–0.261)	0.163 (0.086–0.251)
Women	0.222 (0.140–0.523)	0.155 (0.071–0.261)	0.067 (0–0.228)
HbA1c	*ρ* = 0.000, *p* = 0.999	*ρ* = 0.069, *p* = 0.629	*ρ* = −0.164, *p* = 0.252
Systemic hypertension	*p* = 0.530	*p* = 0.436	*p* = 0.765
Absent	0.222 (0.140–0.427)	0.097 (0.011–0.187)	0.125 (0.048–0.256)
Present	0.301 (0.155–0.523)	0.155 (0.097–0.324)	0.125 (0.015–0.228)
LogMAR VA	—	*ρ* = 0.666, *p* < 0.001	*ρ* = 0.445, *p* < 0.001
Lens status	*p* = 0.134	*p* = 0.148	*p* = 0.851
Phakia	0.301 (0.187–0.582)	0.155 (0.097–0.385)	0.136 (0.030–0.287)
Pseudophakia	0.155 (0.125–0.412)	0.097 (0–0.187)	0.125 (0.024–0.225)
International classification			
Mild NPDR	0.222	0.155	0.067
Moderate NPDR	0.155 (0.155–0.301)	0.097 (0–0.187)	0.125 (0.022–0.222)
Severe NPDR	0.280 (0.155–0.473)	0.140 (0.046–0.242)	0.176 (0.032–0.352)
PDR	0.489 (0.291–0.868)	0.187 (0.084–0.774)	0.125 (0.022–0.222)
Panretinal photocoagulation	*p* < 0.001	*p* < 0.001	*p* = 0.944
Absent	0.155 (0.132–0.281)	0.046 (0–0.147)	0.136 (0.039–0.234)
Present	0.398 (0.179–0.626)	0.187 (0.097–0.398)	0.125 (0.019–0.236)
CSF thickness	*ρ* = 0.043, *p* = 0.740	*ρ* = −0.130, *p* = 0.309	*ρ* = 0.205, *p* = 0.110
Transverse length of disrupted EZ	—	*ρ* = 0.273, *p* = 0.033	*ρ* = 0.375, *p* = 0.003
Transverse length of disrupted ELM	—	*ρ* = 0.437, *p* < 0.001	*ρ* = 0.374, *p* = 0.003
Cystoid macular edema	*p* = 0.246	*p* = 0.212	*p* = 0.920
Absent	0.398 (0.155–0.699)	0.155 (0.097–0.398)	0.032 (0.022–0.301)
Present	0.260 (0.155–0.456)	0.125 (0.046–0.222)	0.146 (0.058–0.234)
Serous retinal detachment	*p* = 0.283	*p* = 0.024	*p* = 0.086
Absent	0.301 (0.155–0.489)	0.155 (0.071–0.372)	0.125 (0.015–0.176)
Present	0.187 (0.155–0.398)	0.097 (0–0.155)	0.234 (0.031–0.301)

**Table 7:** Association between the preoperative transverse length of disrupted EZ (or ELM) and CSF thickness after ranibizumab for DME.

Parameter at baseline	CSF thickness at 12 months	Decrease in CSF thickness at 12 months
Transverse length of disrupted EZ	*ρ* = −0.076,*p* = 0.550	*ρ* = 0.235,*p* = 0.066
Transverse length of disrupted ELM	*ρ* = 0.171,*p* = 0.183	*ρ* = 0.282,*p* = 0.028
